# The combination of single-cell and RNA sequencing analysis decodes the melanoma tumor microenvironment and identifies novel T cell-associated signature genes

**DOI:** 10.7150/jca.96484

**Published:** 2024-08-06

**Authors:** Sihan Luo, Daiyue Wang, Jiajie Chen, Shaocheng Hong, Yuanyuan Fang, Lu Cao, Liang Yong, Shengxiu Liu

**Affiliations:** 1 Department of Dermatology, First Affiliated Hospital of Anhui Medical University, Hefei, Anhui, 230022, China.; 2 Key Laboratory of Dermatology (Anhui Medical University), Ministry of Education, Hefei, Anhui, 230022, China.; 3 Inflammation and Immune-Mediated Diseases Laboratory of Anhui Province, Hefei, Anhui, 230022, China.; 4 Laboratory of Stem Cell, The First Affiliated Hospital of Ningbo University, Ningbo, Zhejiang 315010, P R China.; 5 Department of Gastroenterology, The First Affiliated Hospital of Anhui Medical University, Hefei, 230032, China.; 6 Department of Obstetrics and Gynecology, The Second Hospital of Anhui Medical University, Hefei, China.; 7 Department of Dermatology, The First Affiliated Hospital of Ningbo University, Ningbo, Zhejiang, China.

**Keywords:** T cell, single-cell sequencing, melanoma, tumor microenvironment, PEB4B

## Abstract

Skin cutaneous melanoma (SKCM), a malignant melanocyte-derived skin cancer, potentially leads to fatal outcomes without effective treatment. The variability in immunotherapy responses among melanoma patients is significantly influenced by the intricate immune microenvironment, particularly due to the status of tumor T cells, encompassing their activity, exhaustion levels, and antigen recognition capabilities. This study utilized single-cell RNA sequencing (scRNA-seq) to analyze 34 melanoma samples from two public datasets (GSE215120 and GSE115978). Herein, we extracted 706 marker genes associated with immune checkpoint (ICP) therapy from these T cells, 509 markers of T cells from 11 melanoma tissues, and eventually identified 33 candidate genes. These genes underwent LASSO and COX regression analyses to identify the signature genes. Of the initial 33 candidate genes, we successfully isolated six distinct T cell-associated immunotherapy-related genes (IRTGs). Additionally, the computation of each patient risk score proved beneficial in evaluating the immune cell infiltration level and functions as an independent prognostic factor for melanoma patient survival. The risk score results revealed promising predictive outcomes in determining the response of melanoma patients to immunotherapy. Notably, our study is the first to reveal the potential correlation between signature gene PEB4B and the immune microenvironment in melaoma, which was explored with multiple immunofluorescence (IF) and Immune Infiltration Assessment. In a conclusion, our findings demonstrate the potential utility of a risk score dependent on signature genes as a predictive tool for assessing the prognosis and response to immunotherapeutic interventions in melanoma patients.

## Introduction

Skin cutaneous melanoma (SKCM) originates from the malignant conversion of melanocytes located in the basal layer of the skin epidermis^1^, usually caused by exposure to natural sunlight and ultraviolet radiation^2^. The SKCM represents the most lethal skin cancer type, and its occurrence is elevatings globally, especially among White populations^3^. The main treatment option is surgery combined with immunotherapy, but there is a high risk of relapse for thicker melanomas and patients with local lymph node involvement^4^. The immune response in the tumor microenvironment (TME) is essential in determining tumor invasion, development, and how the tumor responds to immunomodulators. Extensive research has been conducted on SKCM prognostic biomarkers, particularly focusing on the tumor-infiltrating immune cell density and type as well as the immune gene expression levels^5^. In addition, utilizing immune-related genes or tumor-infiltrating immune cell characteristics has granted great value in predicting recurrence and prognosis in SKCM patients^6^, ^7^.

Moreover, T cells substantially contribute to tumor suppression and elimination and influence their prognosis and progression^8^, ^9^. Cancer-associated fibroblast (CAF) has been shown to inhibit cytotoxic T lymphocyte (CTL) activity through arginase in melanoma^10^. Furthermore, Zhang *et al.* have conducted single-cell RNA sequencing (scRNA-seq) analysis for 11 melanoma patients, revealing the gene expression levels in T cells, CAFs, and malignant cells. Remarkably, these samples included pre- and post-immunotherapy tumor patients, who provided genetic changes in T cells before and after immunotherapy^11^.

Immune checkpoint inhibitors (ICIs) have revolutionized the therapeutic landscape for many cancers, especially melanoma^12^. Immunotherapy with many ICIs targeting programmed cell death protein 1 (PD-1), PD-1 ligand (PD-L1), and CTL antigen-4 (CTLA-4) has significantly increased the clinical outcomes of melanoma patients^13^. This therapeutic strategy, which is central to the management of melanoma, employs immune checkpoint (ICP) inhibition to stimulate T-cell-mediated tumor elimination, representing a pivotal advancement in the field^14^. Additionally, Jerby-Arnon *et al.* have conducted scRNA-seq analysis for 33 melanoma tumors, identifying a malignant cell resistance program in melanoma patients to promote immune escape, leaving patients unable to benefit from ICIs^15^. Notably, this malignant cell program was related to T-cell exclusion and was predictive of ICI resistance. Therefore, exploring its predictive role in the prognosis of melanoma patients is possible.

This study first screened 33 immunotherapy-related genes in in the T cells (IRTGs) of melanoma tumors that were highly associated with immunotherapy response. From the list of candidate genes, we further identified six signature genes and constructed a prognostic signature to generate a predictive risk score, which was strongly correlated with immunotherapy response and prognosis. Then, the prognostic model was confirmed to serve as an independent prognostic factor for melanoma patients.

## Methods and Materials

### Collection of data for Single-cell analysis

A total of 7 acral and 4 cutaneous melanoma RNA sequencing data were obtained from the public dataset provided by Zhang *et al.* These processed single-cell/bulk RNA data are accessible at the GEO database with the accession number GSE215121^11^. The samples included one patient with CM1 and CM1-lym as well as one with AM3-pre and AM3-post tissues. Additionally, we incorporated 23 samples from GSE115978, comprising 13 pre-immunotherapy and 10 post-immunotherapy specimens^15^.

### Identifying cell clusters of scRNA-seq

The gene expression in each cell was calculated relative to the gene multiplied by 10,000 using the natural log transformation applied using the log(x+1) technique. The resulting normalized expression matrix was used to determine the top 2000 highly variable genes (HVGs). Subsequently, the genes underwent scaling before conducting a principal component analysis (PCA). The R Harmony package was employed to eliminate batch effects, utilizing the top 30 PCA components. Utilizing harmonized data, the k-nearest neighbors (KNN) algorithm was employed to calculate distances, subsequently leading to the construction of a shared nearest neighbor (SNN) graph. In order to perform cluster recognition, the modular function was modified according to the clustering algorithm that was used. The clusters obtained were then visualized on a two-dimensional map generated employing uniform manifold approximation and projection (UMAP).

The "FindAllMarkers" function was employed to find each cluster marker gene, employing the subsequent parameters: logfc.threshold = 0.25, min.pct = 0.25, and min.diff.pct = 0.25. The DotPlot and featureplot tools from the Seurat package were employed to represent these marker gene expression patterns visually across clusters. The annotation of cell clusters was performed employing the previously reported DEGs and well-recognized cellular markers^11^. Furthermore, to explore the heterogeneity of melanoma cells, re-clustering was performed.

### Cell-cell communication

Our study utilized the CellChat package to infer cell-cell communication across all cell types using scRNA-seq data^16^. The prediction of cell-cell interactions between the various cell types was established with a significant threshold of 0.05 (P-value).

### CNV evaluation of melanoma cells

The InferCNV package was used to deduce CNVs in melanoma cells and identify cancerous cells using the default settings. Genes having an average count of < 0.1 between all cells were excluded before analysis. The calculation of the CNV score follows the previous methodology^17^. The melanoma cells were categorized into two groups, namely low and high scores, based on their accumulation scores of CNV.

### GO and KEGG enrichment analyses of DEGs

Herein, we conducted a comparative analysis of the upregulated and downregulated genes with respect to the associated terms in the GO (http://www.geneontology.org/) and the KEGG databases. These analyses aimed to ascertain the functional and pathway significance of the DEGs among high- and low-CNV subtypes. In addition, the KEGG analysis was also performed in the 33 candidate genes (metascape; https://metascape.org/gp/index.html).

### Gene set enrichment analysis (GSEA) and GSVA

The inquiry into gene function was conducted using the GSEA program and the MSIGDB database obtained from the GSEA website (http://software.broadinstitute.org/gsea/msigdb). The process of differential gene induction was used to rank pathways, and GSEA was conducted employing the Pi package and MsigdbH. The GSVA package was employed to assign estimates of pathway activity to particular cells.

### Scenic analysis

The SCENIC tool was employed to leverage scRNA-seq data for rebuilding gene regulatory networks and ascertaining stable cell states. The analysis was conducted using the pySCENIC package in Python (version 3.8), wherein the enrichment of transcription factors and the regulons activity were evaluated^18^. The gene regulatory network was constructed employing co-expression and DNA motif analysis. The cell state identification included examining the network activity occurring inside each cell. To establish the search space for transcription factor regulatory networks around the transcription start site, the gene motif ranking within a 10 kb radius was employed as a guiding parameter. The human gene-motif rankings were obtained from https://resources.aertslab.org/cistarget/.

### Pseudo-temporal ordering of CAFs

The Monocle package was employed to analyze the pseudotime trajectories of CAFs. Monocle uses pseudo-temporal profiling of scRNA-seq data to detect cellular changes throughout the CAF differentiation. The raw UMI counts, along with their clustering information, were incorporated into the "newCellDataSet" function and subsequently transformed into a reduced dimensional space employing the discriminative dimensionality reduction with trees (DDRTree) technique, a contemporary manifold learning approach. Then, the CAFs were arranged based on their pseudo-time.

### Construction and assessment of the prognostic IRTs_score model

The glmnet R package was deployed to conduct LASSO and multiple COX regression analyses on 33 candidate genes to identify signature genes that may accurately predict the melanoma patient prognosis. A predictive IRTs_score model was constructed using these signature genes.

IRTs_score was evaluated as follows:

IRTs_score = Σ (Expi * coefi)

where Expi and Coefi represent each gene expression and the corresponding risk coefficient, respectively.

### KM survival curves of high and low-risk melanoma patients

Bulk transcript data of the TCGA-SKCM cohort were obtained by accessing the TCGA database (https://portal.gdc.cancer.gov/), and melanoma cohort of GSE54467, GSE65904, GSE22153, and GSE59455 were acquired from the GEO database. Depending on the risk scores, patients were categorized into high- and low-risk groups. The initial steps involved the construction of KM survival curves to evaluate the discrepancy in survival outcomes. The survival analysis was conducted using the survival package (version 3.4.0) and the survminer package (version 0.4.9), enabling the identification of signature genes exhibiting contrasting survival rates between the high- and low-risk groups.

### Comparison of immune condition between high- and low-risk groups

The CIBERSORT algorithm was utilized to quantify the immune cell infiltration extent in melanoma tissues, subsequently leading to an examination of the immune cell infiltration and risk score correlation. Furthermore, a comparison was conducted between immune cell infiltration and six signature genes. Additionally, immune-related scores, such as stromal, immune, and ESTIMATE scores, were compared between both risk groups.

### Differences in ICP gene expression and IPS between both risk groups

The Wilcoxon signed-rank test was employed for comparing the ICP gene expression between both groups. Various types of immunotherapy management, including PD-1/PD-L1/PD-L2 and CTLA-4 blockers, were predicted by IPS in patients. Additionally, the iMvigor210, PRJEB25780, PRJEB23709, and GSE35640 cohorts were used to calculate values for complete response (CR)/partial response (PR) and SD/PD.

### The scRNA-seq analysis of the six signature genes

The analysis of scRNA-seq was performed on ten cohorts (GSE115978, GSE120575, GSE123139, GSE134388, GSE139249, GSE148190, GSE159251, GSE166181, GSE179373, and GSE72056) obtained from the GEO database. Moreover, we used the TISCH2 (https://tisch.comp- genomics.org/home/) to visualize the six signature gene expression in various single-cell transcriptome datasets.

### Melanoma tissue microarray and multiple immunofluorescence staining (IF)

The 44 human melanoma and 4 normal skin tissue microarrays (Cat No. ZL-MEL962) were acquired from ShangHai Zhuoli Biotech Company (China). The slides were processed using the following sequence: immersed in 100% xylene for two 10-min intervals, immersed in 100% ethanol for two 3-min intervals, immersed in 95% ethanol for two 2-min intervals, immersed in 70% ethanol once, and rinsed with distilled water twice. Moreover, we conducted the antigen retrieval by subjecting the sample to boiling in a citrate pH 6 (Dako) solution for 10 min and cooled to room temperature (RT). Then, the slides were cleansed with TBST (1 x TBS 0.1% Triton-X) and subjected to blocking in 1% NDS solution diluted in TBST for 1 h at RT and then incubated. Subsequently, the slides were exposed to primary antibodies overnight at 4 °C followed by being removed by washing the slides utilizing TBST for 5 min 4 times. The secondary antibodies, labeled with fluorophore gating, were diluted in a solution containing 1% NDS and went through incubation with the slides for 1 h at RT. Nuclei were treated with DAPI (1 μ g μ L-1). The slides were washed four times with TBST before mounting and imaging.

## Results

### Workflow of the present study

### Construction of scRNA-seq atlases of melanoma

The results identified 28 distinct cell clusters, including immune, stromal, and melanoma cells (**Figure 2A**). Further analysis revealed that (**Figure 2B**) the immune cells could be categorized into T cells (IL7R, CD8B/3D, TRAC, and NKG7), B cells (CD79A, BANK1, and MS4A1), and monocytes (LYZ and CD14/68). Additionally, the stromal cells were classified into endothelial cells (PECAM1 and VWF) and fibroblasts (COL1A2/3A1). Notably, these cell cluster compositions, particularly the melanoma cells and T lymphocytes, were distinct in different melanoma samples (**Figure 2C**). Subsequently, we examined differentially expressed genes (DEGs) across various cell types and labeled markers specific to each cell type. (**Figures 2D-E**).

**Figure 1:**
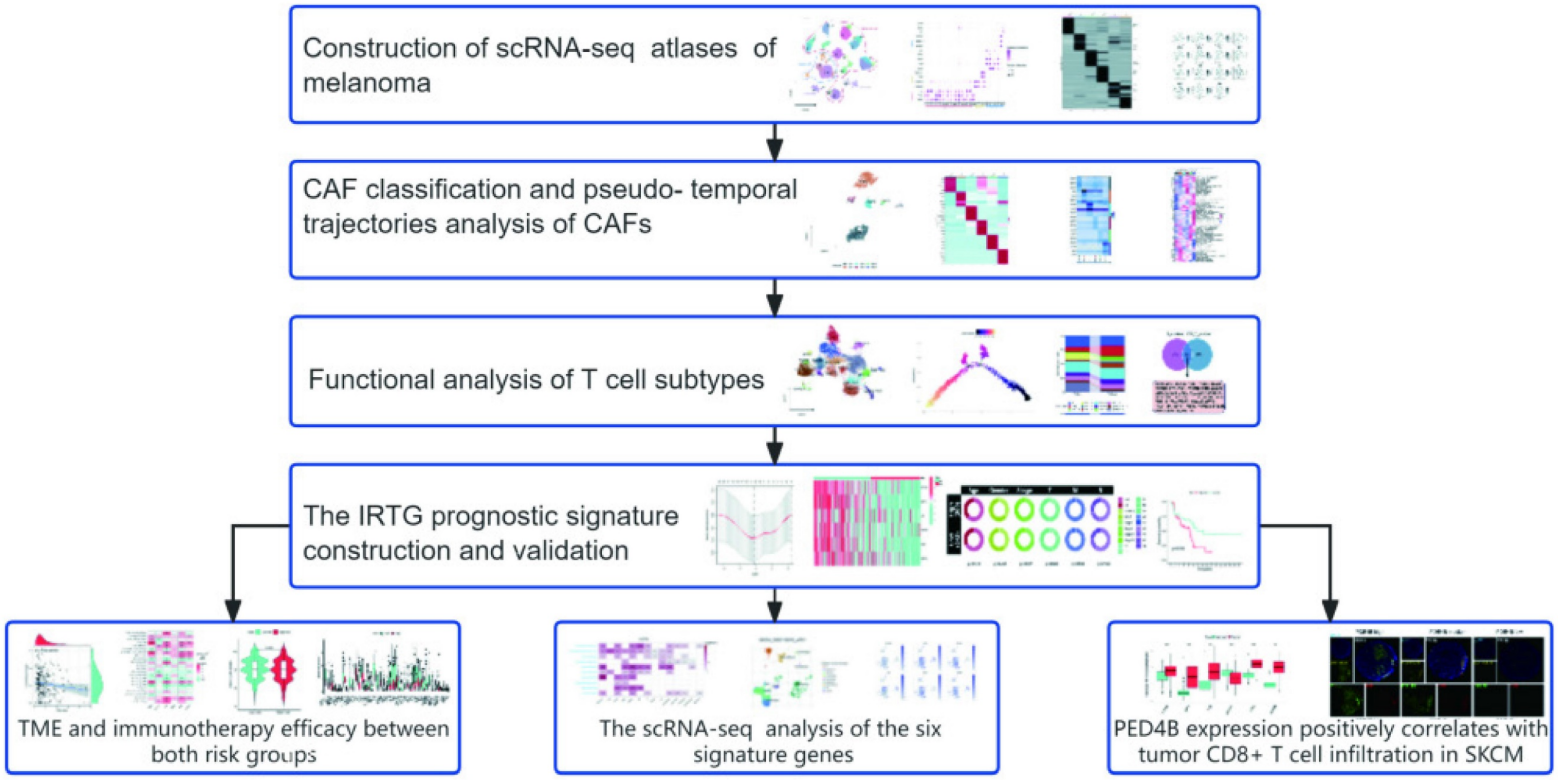
Workflow of the present study

**Figure 2:**
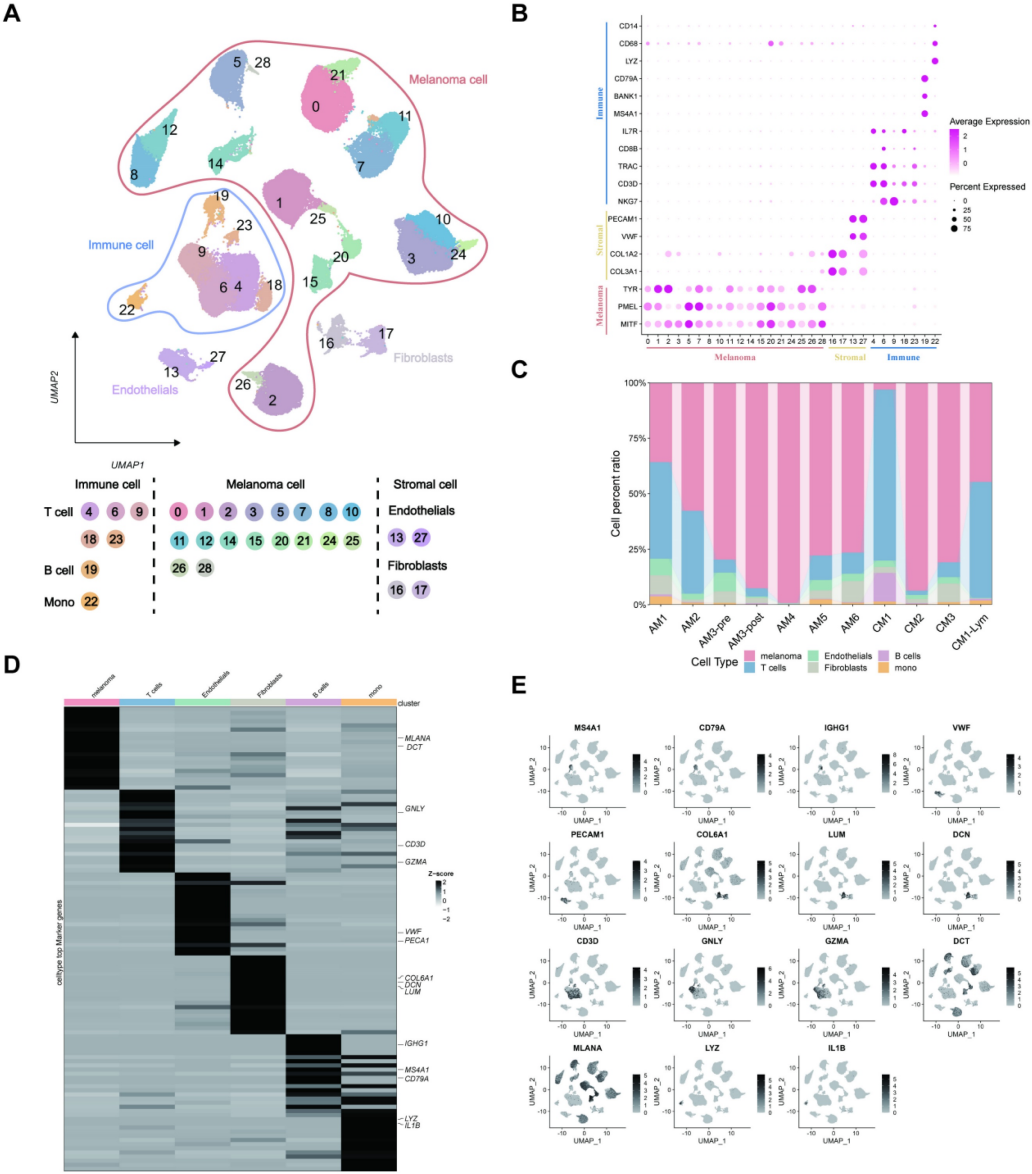
The scRNA-seq profiling of the melanoma environments. (A) The 2D plots of UMAP dimensionality reduction of the melanoma, stromal, and immune cells. (B) A dot plot showing the expression levels of marker genes associated with cell subtypes within the melanoma environments. (C) A proportion chart showing the distribution of cell clusters and subtypes across tissues, with the x-axis representing 11 different patients. (D) The heatmap displaying the marker gene expression in the six cell subtypes. (E) The UMAP plots showing the differential marker gene expression among melanoma cell subtypes.

Following the classification of melanoma samples at the cellular level, our objective is to establish a communication network between subtypes of melanoma, immune, and stromal cells. The comprehensive outcomes of the CellChat analysis are visually represented in Figure 2 through Sankey diagrams, dotplot, and chordal graphs. Subsequent examination of incoming communication among these cells unveiled a shared pattern between stromal and melanoma cells** (Supplement Figures 1A-B)**. Furthermore, **Supplementary Figures 1C-E** provide detailed insights into potential molecular interactions. We can observe functional connections between these clusters of cells.

### Copy number variation (CNV) analysis of melanoma cell subtypes

**Figure 3:**
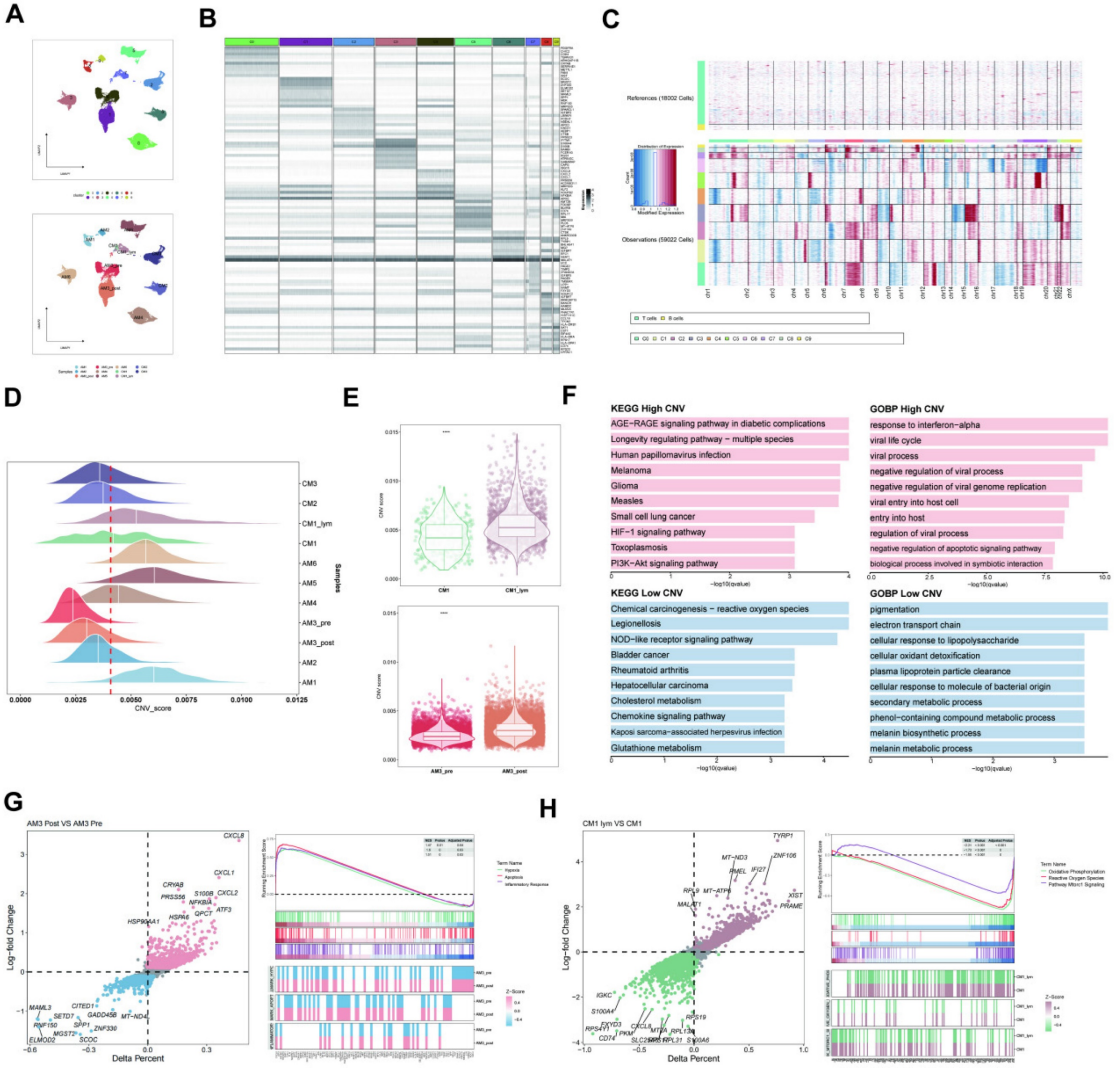
Inferred CNV analysis of melanoma cells. (A) Melanoma cell clusters differentiation. (B) The top ten marker genes for each melanoma cell cluster. (C) The heatmap depicting the extensive CNV in melanoma cells. Red signifies a significant amount of CNV, whereas blue indicates a modest CNV level. (D) The classification of melanoma cells into several groups according to their CNV score. Joyplots showing the dispersion of CNV scores across several specimens. Red dashed lines show the threshold values. (E) The comparison of the CNV scores between the CM1 and CM1-lym as well as AM3-pre and AM3-post. (F) The KEGG and GO analysis for the upregulated and downregulated 100 DEGs between the high- and low-CNV samples. (G-H) The Δ percent of cells and log-fold change calculated depending on the Wilcoxon rank-sum test findings for DEGs between the CM1 and CM1-lym as well as AM3-pre and AM3-post. The represented GSEA results for the DEGs.

The melanoma cells were divided into ten clusters using cluster analysis and were visualized according to the tumor samples (**Figure 3A**). Moreover, we visualized the top ten marker genes for each melanoma cell cluster (**Figure 3B**). Subsequently, the CNV status from various cell types was determined utilizing T and B lymphocytes as reference controls using InferCNV analysis (**Figure 3C**). Consistent with the findings of Zhang *et al.* in pre- and post-treatment samples of a single patient who received immunotherapy, variations in CNV were found on chromosome 4. The CNV observed in tumor specimens displayed significant heterogeneity, with varying degrees of CNV accumulation among various patients and tissue types (**Figure 3D**). Subsequently, we categorized all melanoma cells into high- and low-CNV groups. Compared with cutaneous melanoma (CM1) and melanoma cells derived from cutaneous melanoma lymphatic metastasis tissues (CM1-lym) as well as pre- (AM3-pre) and post-immunotherapy acral melanoma tissues (AM3-post), CM1-lym and AM3-post tissues exhibited significantly elevated CNV levels, suggesting a more aggressive phenotype (**Figure 3E**). The Gene ontology (GO) and Kyoto Encyclopedia of Genes and Genomes (KEGG) analyses for the DEGs between both CNV tumor samples showed the related pathways and functions (**Figure 3F**). **Figure 3G-H** depicts that differences in gene expression patterns were significant between CM1 and CM1-lym as well as AM3-pre and AM3-post for melanoma cells.

### CAF classification and pseudo-temporal trajectories analysis of CAFs

**Figure 4:**
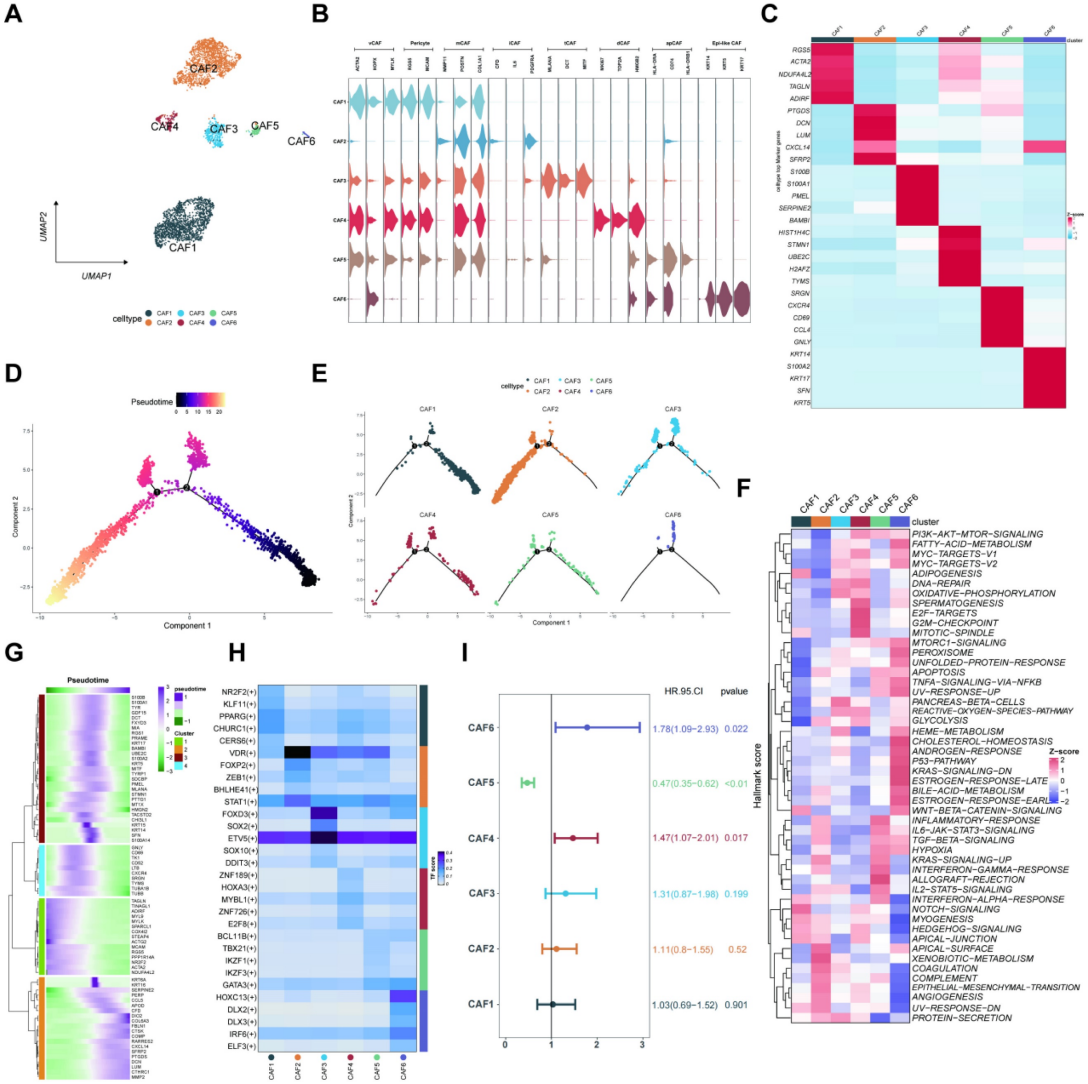
CAFs heterogeneity in melanoma. (A) The UMAP plots displaying the identification of CAF subtypes. (B) The violin plots depicting the differential expression of marker genes of CAF subtypes. (C) The heatmap illustrating marker genes of CAF subtypes based on the Z-score. (D-E) Monocle predicting the trajectory of CAF subtype differentiation. (F) Comparing the enrichment of hallmark pathways among CAF subtypes through GSVA. (G) Heatmap depicting the marker gene expression of CAF subtypes in different branches, as annotated into four major clusters. (H) The transcription factors for the CAF subtypes. (I) The univariate Cox regression analysis for the six CAF subtypes.

The CAFs were classified into six distinct cell subtypes (CAFs1-6) depending on biological functions, cellular interactions, marker genes, and spatial distribution in the TME. These subtypes included vascular CAF (vCAF), pericyte, matrix CAF (mCAF), inflammatory CAF (iCAF), tumor-like CAF (tCAF), dividing CAF (dCAF), antigen-presenting CAF (apCAF), and epithelial-like CAF (epi-CAF) (**Figures 4A-B**)^19^. **Figure 4C** shows the respective marker genes of different CAF subtypes. Subsequently, we found that the differentiation trajectory of different CAF cell subtypes was different through pseudo-temporal trajectories analysis (**Figures 4D-E**). Then, we applied gene set variation enrichment analysis (GSVA) to reveal CAF functions, demonstrating the related pathways of CAFs (**Figure 4F**). Besides, the markers of the four reclassified CAF subtypes based on the pseudotime trajectories analysis were shown in heatmaps (**Figure 4G**). The main transcription factors for the CAF subtypes were evaluated (**Figure 4H**). A multivariate COX regression analysis for the CAFs1-6 identified the risk factors depending on the individual gene expression levels (**Figure 4I**).

### Functional analysis of T cell subtypes

**Figure 5:**
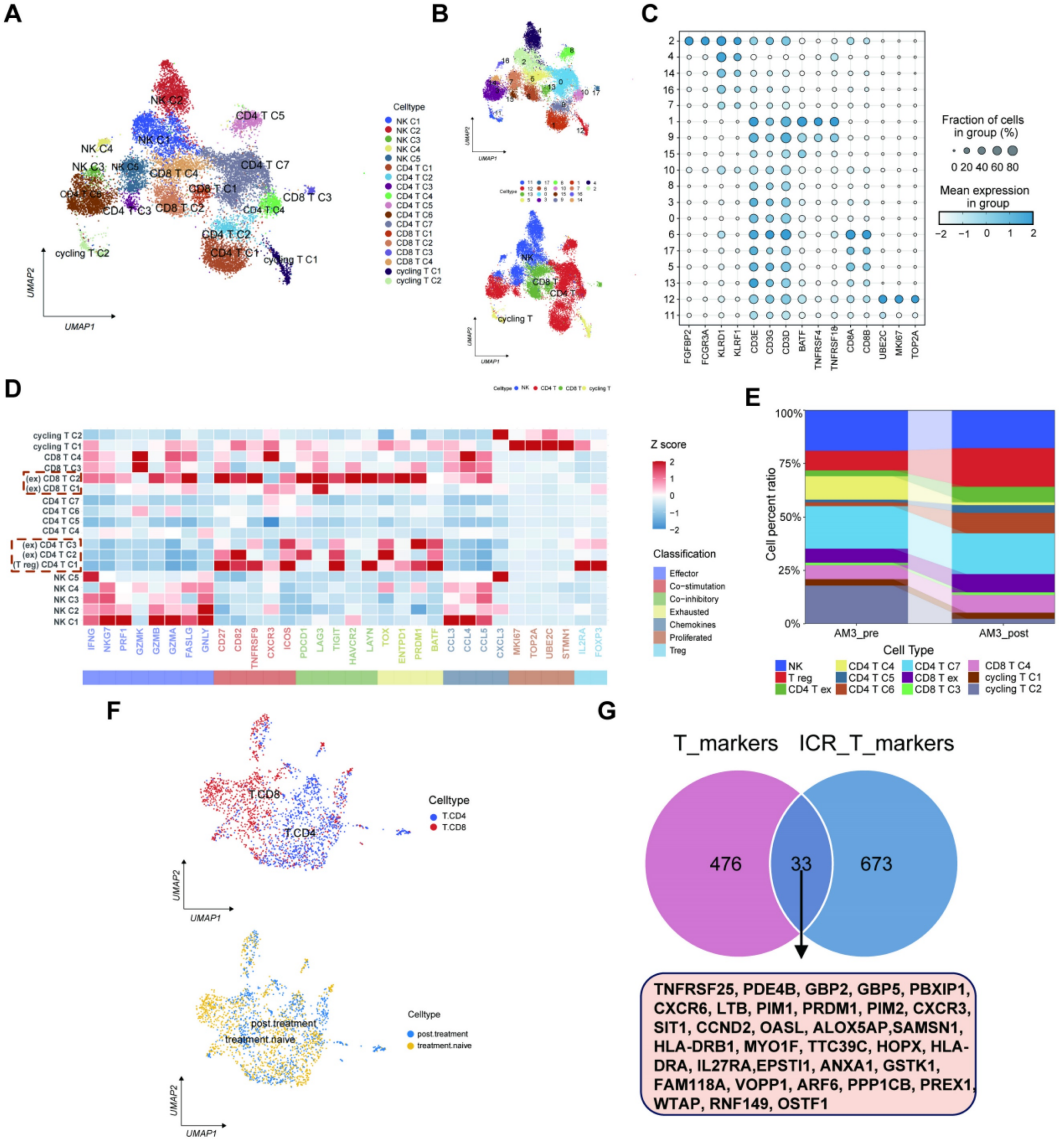
Functional analysis of T cell subtypes. (A-B) The UMAP plots displaying the identification of T cell subtypes and clusters, encompassing CD4/8 T cell and NK cell subtypes. (C) Dot plot manifesting T cell subtypes marker genes. (D) The major gene expression in T cells in melanoma samples based on Z-score. (E) The histogram illustrating the relative abundance of T cells inside the tumor tissue of each patient under analysis. (F) The UMAP plots showing the identification of CD4/8 T cell subtypes from the GEO database for 33 melanoma patients with immunotherapy resistance (GSE115978). (G) Identification of 33 IRTGs.

Herein, we also focused on the immune cell subtypes and classified them into five groups of natural killer (NK) cells, seven groups of CD4 T cells, four groups of CD8 T cells, and two groups of cycling T cells depending on the marker gene expression levels (**Figures 5A-C**). Moreover, we used Z-score to show the expression of major genes in T cells in melanoma samples (**Figure 5D**). The percentage of T cell clusters between AM3-pre and AM3-post tissues was represented on a proportion chart (**Figure 5E**). The scRNA-seq data of gene expression levels in T cells were collected from the GEO database for 33 melanoma patients with immunotherapy resistance (GSE115978). Furthermore, we divided the immunotherapy-related T cells into two clusters: CD4/8 T cells (**Figure 5F**). Meanwhile, we extracted 706 marker genes associated with ICP therapy from these T cells, 509 markers of T cells from 11 melanoma tissues, and eventually identified 33 IRTGs (**Supplementary Table 1; Figure 5G**).

### The IRTG prognostic signature construction and validation

**Figure 6:**
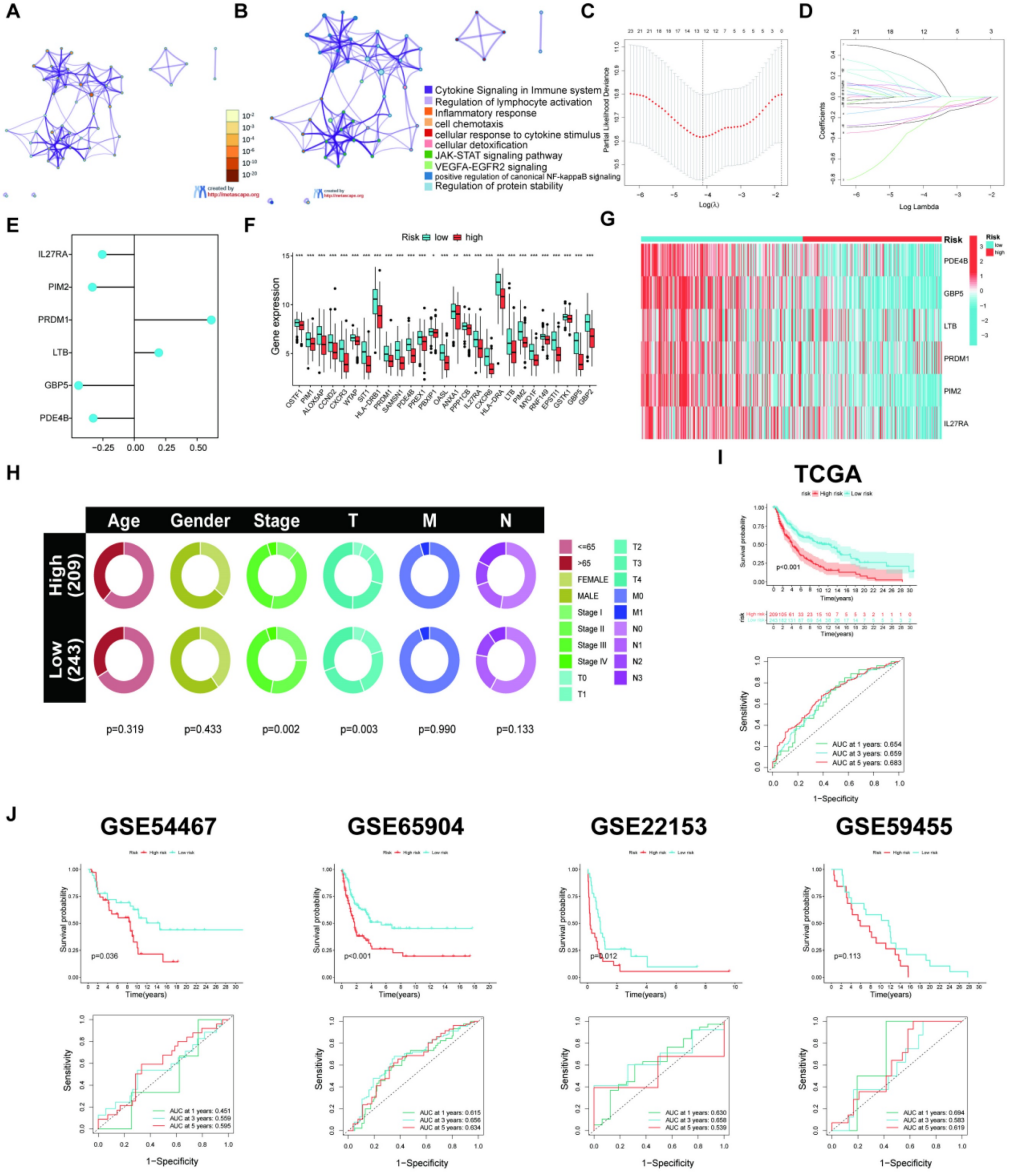
Construction of the prognostic signature. (A-B) The KEGG analysis for the 33 candidate genes. (C-D) LASSO regression analysis and the partial likelihood deviation for the 33 genes. (E) Forest plot of the multivariate Cox regression analysis for the 6 genes. (F) Variations in levels of ICP gene expression in the two risk groups. (G) The heatmap displaying the six signature gene expressions in the two risk categories within the training cohort. (H) Risk score and survival outcome of each sample. (I) Pie charts representing the Chi-squared test of clinicopathologic factors in high- and poor-risk groups. (J) KM and ROC curves showing the prognostic value in multiple cohorts.

The results indicated 33 prognostic genes mainly enriched in some immune-related pathways using a KEGG analysis (**Figures 6A-B**). The LASSO and COX regression analyses were conducted for these 33 genes to identify six signature genes: IL27RA, PIM2, PRDM1, LTB, GBP5, and PDE4B (**Figures 6C-E**). The risk score was determined depending on the signature gene expression levels alongside their corresponding risk coefficient value as follows: [IL27RA expression level × (-0.254915516014614)] + [PIM2 expression level × (-0.333557686339427)] + [PRDM1 expression level × (0.61377881693067)] + [LTB expression level × (0.195208657137769)] + [GBP5 expression level × (-0.443593637428344)] + [PDE4B expression level × (-0.326705622270952)]. Depending on the risk score, 469 SKCM patients acquired from the TCGA database were allocated into high- and low-risk groups. The low-risk groups had higher ICP gene expression levels (**Figure 6F**) as well as overexpressed IL27RA, PIM2, PRDM1, LTB, GBP5, and PDE4B, indicated by a heatmap (**Figure 6G**). Furthermore, the high-risk group exhibited a heightened death incidence (**Figure 6H**). Multivariate COX regression analysis elucidated that age, sex, tumor stage, and risk score could serve as independent predictive factors (**Figure 6I**). The Kaplan-Meier (KM) curves for the training cohort from TCGA manifested that low-risk patients had a significantly extended overall survival (OS; p < 0.001), with 1-, 3-, and 5-year AUC values of 0.654, 0.659, and 0.683, respectively (**Figure 6J**). The outcomes from the four validation cohorts, GSE54467 (p = 0.036, 1-, 3-, and 5-year AUC = 0.451, 0.559, and 0.595, respectively), GSE65904 (p < 0.001, 1-, 3-, and 5-year AUC = 0.615, 0.656, and 0.634, respectively), GSE22153 (p = 0.012, 1-, 3-, and 5-year AUC = 0.630, 0.658, and 0.539, respectively), and GSE59455 (p = 0.113, 1-, 3-, and 5-year AUC = 0.694, 0.583, and 0.619, respectively), indicated that patients with a low-risk score experience longer disease-free survival (DFS).

### TME and immunotherapy efficacy between both risk groups

**Figure 7:**
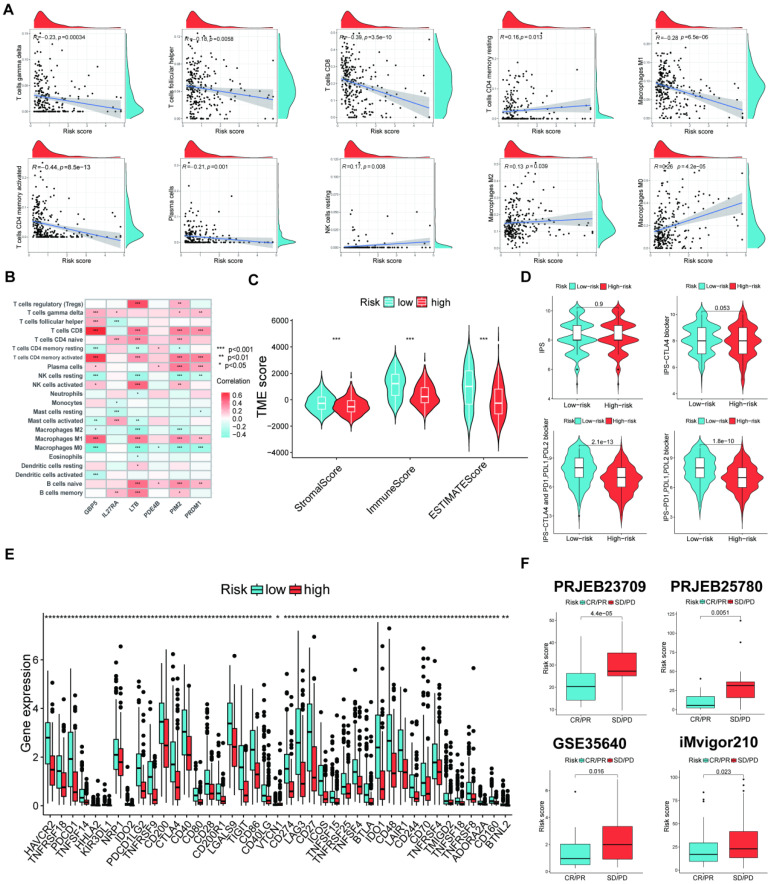
Assessment of TME in high- and low-risk groups. (A) Correlation between risk score and different immune cell types. (B) Correlation between the abundance of immune cells and six signature genes. (C) Comparison of immune-related scores between the two risk groups. (D) The immunotherapy responses of the two risk groups. (E) The ICP gene expression levels between the two risk groups. (F) Patients who achieved CR/PR had significantly reduced risk ratings than patients with SD or PD in all four cohorts. (*p < 0.05, **p < 0.01, ***p < 0.001)

The study revealed a direct correlation between risk scores and M2/M0 macrophages and both resting memory CD4 T cells and NK cells. Conversely, an adverse association was found between risk scores and γδ T cells, follicular helper T cells, CD8 T cells, activated memory CD4 T cells, plasma cells, and M1 macrophages (**Figure 7A**). Furthermore, the six signature genes were related to several instances of immune cell infiltrations (**Figure 7B**). **Figure 7C** shows that the low-risk group exhibited elevated stromal and immunological scores. Further, the low-risk group that underwent various types of ICP inhibition treatment had significantly increased immunophenoscores (IPSs; Figure 7D), indicating a more favorable immunotherapeutic response. The low-risk group had increased ICP gene expression levels (**Figure 7E**), suggesting a possibility for enhanced responsiveness to immunotherapy. Moreover, we further confirmed the risk score effectiveness in the prediction of ICI responses in the PRJEB25780, iMvigor210, PRJEB23709, and GSE35640 cohorts. Patients with stable disease (SD)/progressive disease (PD) had a lower risk score than those with high risk (**Figure 7F**).

### The scRNA-seq analysis of the six signature genes

**Figure 8:**
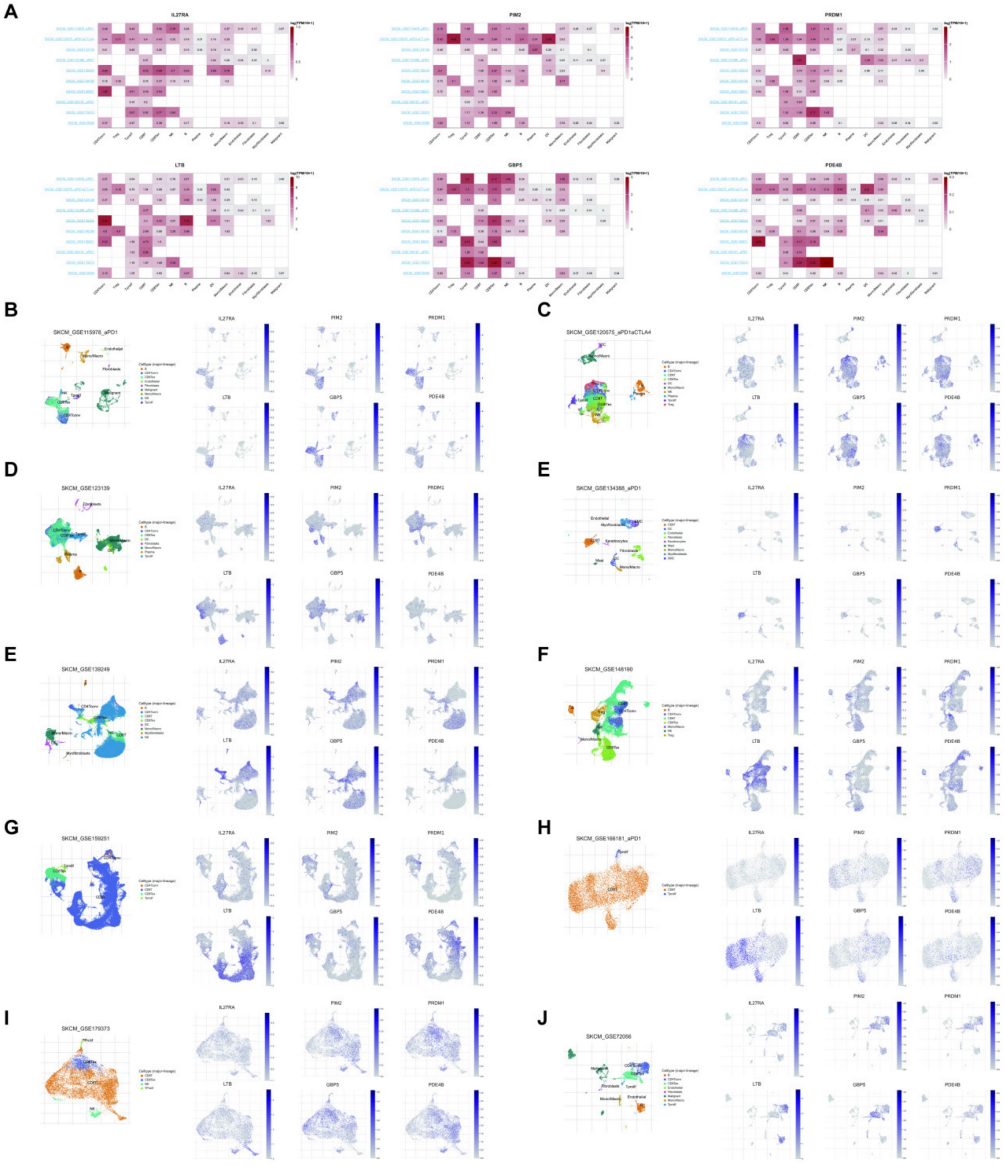
(A) The levels of six signature gene expressions in many immune cell types in ten SKCM immunotherapy-related scRNA-seq datasets. (B-J) The expression location of the six signature genes in ten immunotherapy-related scRNA-seq datasets.

Herein, we observed the six signature gene expression levels in many immune cell types in ten SKCM immunotherapy-related single-cell sequencing datasets. The six signature genes were mainly expressed in immune cells compared to stromal cells (**Figure 8A**). Furthermore, **Figures 8B-J** show the signature genes significantly expressed in the CD4/8 T cells.

### PED4B expression positively correlates with tumor CD8+ T cell infiltration in SKCM

**Figure 9:**
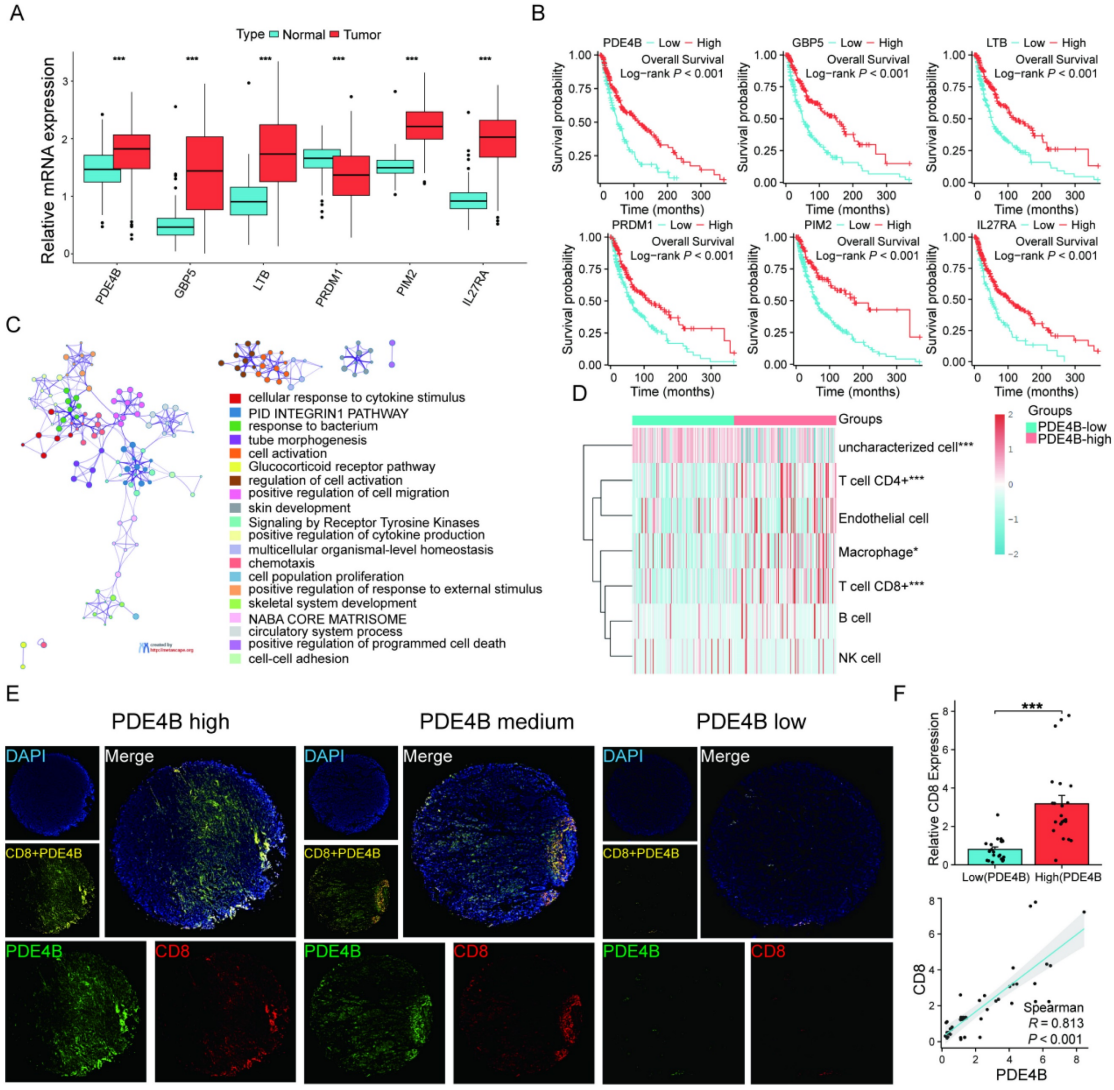
Verification of high PDE4B expression is associated with CD8 +T cell infiltration. (A) Comparison of expression levels of the six signature genes between the SKCM tissues and healthy controls (TCGA and GTEx database). (B) KM curves showing the prognostic value of the signature genes. (C) Enrichment analysis revealing the association between PDE4B high expression and pathways correlated with programmed death in the TCGA-SKCM dataset. Designating the first 25% of PDE4B expression as the high-expression cohort. Categorizing the group with the lowest 25% PDE4B expression as the low-expression cohort. (D) The expression distribution of immune score in high and low PDE4B expression group. The abscissa representing immune cell types, while the ordinate representing the distribution of immune scores among different groups. *p < 0.05, **p < 0.01, ***p < 0.001, asterisks (*) stand for significance levels. (E) Expression of PDE4B and CD8 in the cohort of 44 SKCM detected using immunofluorescence. Representative co-staining images of PDE4B and CD8 in the high, medium, and low PDE4B expression. Scale bars: 500 µm. (F) Relative fluorescence intensity of CD8 between the high and low PDE 4 B expression groups. The correlation between PDE4B fluorescence intensity and CD8 fluorescence intensity detected by immunofluorescence.

Differential expression levels were observed in the six signature genes when comparing SKCM patients to normal controls (**Figure 9A**). Patients exhibiting elevated PED4B expression have a prolonged OS, as the KM analysis shows (**Figure 9B**). Interestingly, pathway analysis based on PDE4B expression elucidated that PDE4B was mainly involved in pathways related to programmed cell death (**Figure 9C**). Combined with the localization of PDE4B in single cells (**Figure 8**), we speculated that PDE4B might be involved in the CD8+ T cells effector function. The TCGA database suggested that high PDE4B expression was accompanied by higher CD8+ T cell infiltration (**Figure 9D**). To validate our conjecture further, we performed multiple immunofluorescence (IF) staining of PDE4B and CD8 using tissue microarrays. Excitingly, there was a co-localized expression of PDE4B and CD8 in tumor tissues (**Figure 9E**), and PDE4B and CD8 expressions were positively correlated (**Figure 9F**). Collectively, our findings indicate that PED4B was correlated with increased CD8 + T cell infiltration in SKCM.

## Discussion

Recently, the incidence of cutaneous melanoma has elevated rapidly^3^. Melanoma patients who have developed distant metastases have a 23% 5-year survival rate, which makes it important to evaluate melanoma prognosis^20^. Immunotherapy is now regarded as a very promising and innovative method for treating metastatic melanoma. The preferred first treatment for this condition is using anti-PD1 antibodies^21^. However, the optimal first therapy for people with advanced melanoma remains uncertain^22^. Not all melanoma patients exhibit sensitivity to immunotherapy, and a subset of them have suboptimal immunotherapy responses and significant adverse effects. These factors impede immunotherapy progress in melanoma treatment^23^. Accordingly, developing a prognostic signature to predict the melanoma patient prognosis and immunotherapy effect is essential. This study collected scRNA-seq data from 63,394 cells from the GEO database of melanoma patients, which consisted of pre- and post-treatment samples obtained from a single patient who underwent immunotherapy^11^, and identified 509 DEGs in the T cells. T cells in cancers have been identified to be related to better outcomes for patients in many human malignant tumors^24^. For example, active CD8+ T cell infiltrations were recognized to be related to improved OS in melanoma patients^25^.

The TME is an intricate ecosystem consisting of several interdependent cell populations^26^. The cell populations consist of diverse infiltrating immune, stromal, and tumor cells^26^. The constitution of the TME has implications for the advancement and spread of tumors, the immune response to tumors, and the effectiveness of therapeutic interventions^27^-^29^. Fibroblasts are the main components of tumor stromal cells. Additionally, CAFs are a diverse group of cells with multiple roles in TME^30^. Therefore, to understand interactions among cell populations and the impact of individual cells on patient prognosis, it is crucial to study tumor cell populations through scRNA-seq data. Our study demonstrated the scRNA-seq atlases of 11 melanoma tumors by identifying the T cells, CAFs, and malignant cells. In addition, we found that cell populations would influence and connect with each other through CellChat analysis. Besides recognizing DEGs in T cells, we also collected 706 immunotherapy-related genes by identifying the marker genes in T cells from the GEO database (GSE115978)^15^. Moreover, we screened 33 candidate genes by combining 509 DEGs and 706 immunotherapy-related genes.

We identified six signature genes by performing Lasso and Cox regression analysis for the 33 candidate genes. These particular methods were chosen due to their ability to handle high-dimensional data and their applicability in survival analysis, respectively. The selected genes are known to have significant involvement in immune response, making them potential predictors for immunotherapy outcomes.

Moreover, we can calculate the risk scores of individuals based on the expression levels of signature genes and the risk coefficient, which called immunotherapy related genes in T cells (IRTGs) prognostic model. The model can be used to forecast the prognosis and immunotherapy responses of melanoma patients. The melanoma patients in various datasets, including TCGA, GSE54467, GSE65904, GSE22153, and GSE59455, can be divided in high- and low- risk groups according to the IRTs risk scores. This risk stratification has the potential to transform treatment decision-making processes by allowing clinicians to tailor therapies based on an individual's predicted response to immunotherapy. In addition, we validated the prognostic value of IRTs risk scores for comparing the survival status and immunotherapy responses between the patients with different risk scores. The prognosis and immunotherapy effect of melanoma patients with high-risk are worse than those with low-risk, which may be used for guiding clinical stratified treatment. For patients with low-risk, adjuvant chemotherapy and immunotherapy may be considered before and after surgery. For patients with high-risk, overall surgical resection and radiotherapy should be considered, and follow-up examinations should be conducted frequently to monitor recurrence.

In this study, we identified six signature genes, PDE4B, GBP5, LTB, PRDM1, PIM2 and IL27RA. The signature gene PDE4B, higher expressed in patients with more survival, was positive correlated with T cells in melanoma tumors. As we mentioned, T cells in cancers is associated with better outcomes of tumor patients^31^. This suggests that the upregulation of these genes may promote a stronger immune response, potentially leading to improved patient outcomes. Previous studies have found that lung cancer patients with high GBP5 respond better to immunotherapy and have a better prognosis^32^, and we firstly found that GBP5 may be beneficial for patients in SKCM. Interestingly, inhibiting PIM kinases, including PIM2, significantly enhanced the antitumor efficacy of T cells in tumor-bearing mice undergoing Adoptive T cell therapy. This effect was further amplified when combined with anti-PD1 antibody treatment^33^. In addition, the PRDM1 and IL27RA have also been found to affect the prognosis and development of tumors, such as hematological malignancies and hepatocellular carcinoma^34^, ^35^, and their roles in SKCM have been reported for the first time. However, the action of PDE4B have never been reported in tumor, the exploration of PDE4B is innovative. Further research into the role of PDE4B and its relationship with T cell activity could yield novel insights into its potential as a therapeutic target.

It must be noted that there are some limitations to this study. First, the effect of the prognostic signature in this study lacks validation of clinical cases. In addition, this study integrates different datasets for analysis, which may cause a little of deviation. Although the number of samples is limited, the use of single-cell RNA sequencing allows us to analyze thousands of cells per sample. This extensive cellular data provides significant statistical power to our analysis. We recognize that increasing the number of samples would further validate our findings and improve statistical significance, which is an area for future research.

## Conclusions

Overall, we constructed a signature that can stratify risk and predict prognosis and immunotherapy responses in patients with SKCM. Clinicians can develop individualized treatment plans with this signature, especially when selecting patients who could benefit from immunotherapy, which may improve survival of patients. We also found the signature gene PDE4B, higher expressed in patients with more survival, was positive correlated with CD8+ T cells in melanoma tumors.

## Supplementary Material

Supplementary table.
